# Cytokines as Early Markers of Colorectal Anastomotic Leakage: A Systematic Review and Meta-Analysis

**DOI:** 10.1155/2016/3786418

**Published:** 2016-03-09

**Authors:** Cloë L. Sparreboom, Zhouqiao Wu, Adem Dereci, Geesien S. A. Boersema, Anand G. Menon, Jiafu Ji, Gert-Jan Kleinrensink, Johan F. Lange

**Affiliations:** ^1^Department of Surgery, Erasmus University Medical Center, 3015 CN Rotterdam, Netherlands; ^2^Department of Gastrointestinal Surgery, Peking University Cancer Hospital & Institute, Beijing 100142, China; ^3^Department of Surgery, Havenziekenhuis Rotterdam, 3011 TD Rotterdam, Netherlands; ^4^Department of Neuroscience, Erasmus University Medical Center, 3015 CN Rotterdam, Netherlands

## Abstract

*Purpose.* Colorectal anastomotic leakage (CAL) is one of the most severe complications after colorectal surgery. This meta-analysis evaluates whether systemic or peritoneal inflammatory cytokines may contribute to early detection of CAL.* Methods*. Systematic literature search was performed in the acknowledged medical databases according to the PRISMA guidelines to identify studies evaluating systemic and peritoneal levels of TNF, IL-1*β*, IL-6, and IL-10 for early detection of CAL. Means and standard deviations of systemic and peritoneal cytokine levels were extracted, respectively, for patients with and without CAL. The meta-analysis of the mean differences was carried out for each postoperative day using Review Manager.* Results.* Seven articles were included. The meta-analysis was performed with 5 articles evaluating peritoneal cytokine levels. Peritoneal levels of IL-6 were significantly higher in patients with CAL compared to patients without CAL on postoperative days 1, 2, and 3 (*P* < 0.05). Similar results were found for peritoneal levels of TNF but on postoperative days 3, 4, and 5 (*P* < 0.05). The articles regarding systemic cytokine levels did not report any significant difference accordingly.* Conclusion.* Increased postoperative levels of peritoneal IL-6 and TNF are significantly associated with CAL and may contribute to its early detection.

## 1. Introduction

Despite the progress made in surgical techniques and perioperative management, morbidity and mortality after colorectal surgery remain problematic. One of the major causes is colorectal anastomotic leakage (CAL), which contributes to one-third of all postoperative deaths after colorectal surgery [1]. CAL occurs in 3% to 20% [2–4] of patients after colorectal surgery. It is a defect of the colorectal wall at the anastomotic site leading to communication between the intra- and extraluminal compartments [5]. Localized signs such as abdominal pain and postoperative ileus, though being considered as abdominal manifestations of CAL, are very common after colorectal surgery and therefore provide limited diagnostic value [6]. Moreover, systemic manifestations or parameters such as fever, increased leukocyte count, or increased C-reactive protein (CRP) levels are actually also frequently observed and therefore not sensitive in diagnosing CAL [7].

With the current postoperative regimes, CAL is usually confirmed by imaging studies such as endoscopy or CT scan. The median day of diagnosis varies between postoperative days 8 and 13 [8–10]. A recent review shows that more than 50% of CAL was at the highest severity when diagnosed, which requires relaparotomy [11]. This indicates that many early stages of CAL are not diagnosed until progressing to a severe state. So the current regimes seem to be ineffective and insufficient in many cases based on the high rates of invasive reintervention [8, 9]. To this end, methods for early detection of CAL require extensive further exploration.

Occurrence of CAL is a dynamic and progressive process. Before systemic symptoms like fever, leukocytosis, and other septic symptoms become manifest, localized infection at the site of the anastomosis first takes place [12], which involves varying immune cells and cytokines [13]. Some cytokines such as TNF, IL-1*β*, and IL-6 are proinflammatory cytokines that mediate inflammatory response, whereas IL-10 is considered as an anti-inflammatory cytokine modulating the inflammation [14, 15]. Although the surgical trauma also influences levels of these cytokines, abnormal changes of the cytokines still indicate occurrence of the infectious complications including anastomotic leakage. Previous studies have suggested that monitoring cytokine levels in drain fluid or in blood samples may contribute to early detection of CAL, while firm evidence is not available yet. Therefore, this meta-analysis aims to evaluate the value of peritoneal and systemic cytokine levels for early detection of CAL.

## 2. Methods

The methods of this meta-analysis followed the PRISMA (Preferred Reporting Items for Systematic Reviews and Meta-Analyses) statement [16].

### 2.1. Literature Search

The literature search was performed in Medline, Embase, Cochrane, Web of Science, and Google Scholar libraries in August 2014 and updated in July 2015 by two authors. No restrictions regarding publication date or language were applied during the search strategy. The search was restricted to human studies.

### 2.2. Study Selection

Titles and abstracts were screened for relevance by two authors (Zhouqiao Wu and Adem Dereci) independently. All full-text articles evaluating the predictive value of TNF, IL-1*β*, IL-6, and IL-10 in early detection of CAL after colorectal surgery were selected. Articles without a comparison between patients with and without CAL were excluded; reviews, letters to editor, and congress abstracts were excluded as well.

### 2.3. Quality Assessment

Two authors independently judged the quality of included articles using the QUADAS-2 (Quality Assessment in Diagnostic Accuracy Studies) method, which evaluates the risk of bias and the applicability according to four key domains including patient selection, index test, reference standard, and flow and timing [17]. Level-of-evidence was estimated according to Levels of Evidence 2011 from the Centre for Evidence Based Medicine [18].

### 2.4. Data Extraction

Two authors independently extracted means and standard deviations (SD) of cytokine levels of each postoperative day for patients with and without CAL, respectively. Any discrepancies were resolved by reexamination of data until consensus was reached. The mean and SD of cytokine levels per postoperative day were not provided in the articles of Matthiessen et al. [19] and Yamamoto et al. [20]. Primary data of these articles were obtained from the authors themselves. The unit of levels of cytokines was not reported by Fouda et al. in their results [21] but was confirmed according to the methods and the instruction of their ELISA kit [22]. All cytokine data were converted into the same unit ng/mL in the meta-analysis.

### 2.5. Statistical Analysis

Quantitative statistical analysis for binary outcomes was carried out using mean differences with 95% confidence interval. The random-effects model was applied to obtain the 95% confidence interval. Statistical heterogeneity was calculated with the *I* ^2^ statistic, which represents the percentage of variation in study estimates due to heterogeneity, and tested by the Cochran *Q* test (modified *χ* ^2^ test). All statistical analyses were performed using Review Manager version 5.3, the Nordic Cochrane Centre, Copenhagen, Denmark.

## 3. Results

### 3.1. Results of Study Selection and Evaluation

**Figure 1:**
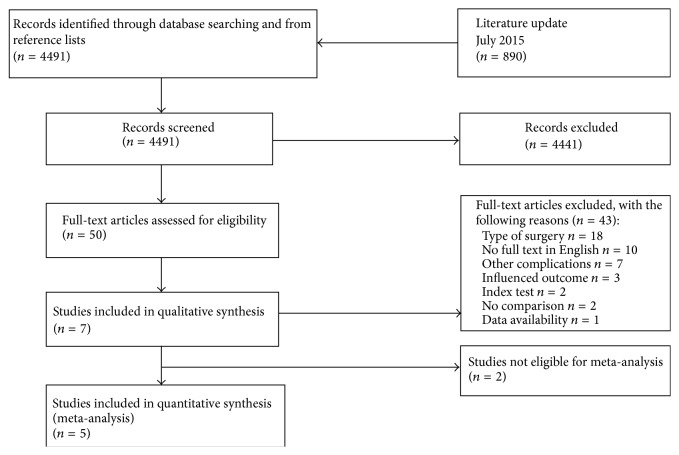
PRISMA flow chart representing selection of articles for review.

Seven articles met final inclusion criteria (Figure 1). All included studies evaluated peritoneal or systemic cytokine levels after colorectal surgery for the diagnosis of CAL (Table 1). All included studies were found to be at high risk of bias while the applicability was considered to be positive (Table 2). The high risk of bias was related to poor patient selection. Furthermore, study designs of included studies led to a low level-of-evidence.

Meta-analysis is a statistical method for pooling the results of several studies reporting similar outcomes in order to gain a better estimate of the effect size of an intervention. It is appropriate to perform a meta-analysis when outcomes are comparable and can be pooled meaningfully. Comparators should be at least similar enough to be combined. All the included studies reported cytokine levels on a similar scale except for the studies of Uğraş et al. [27] and Alonso et al. [28]. The peritoneal cytokine levels reported by Uğraş et al. [27] are approximately 10 to 1000 times higher than the data from the other studies, while the data from Alonso et al. [28] are approximately 50 to 100 times lower than the other inclusions. Despite both studies meeting final inclusion criteria, they were not included in the meta-analysis for peritoneal cytokines.

### 3.2. Definitions of CAL

The definitions of CAL were inconsistent between included studies (Table 3). The studies from Fouda et al. [21] and Bertram et al. [23] based the definition of CAL on clinical signs, mostly focusing on the aspect of drain fluid; the study from Yamamoto et al. [20] included additional imaging studies; the studies from Herwig et al. [24] and Reisinger et al. [26] defined CAL by the necessity of reintervention. The definitions of CAL in the studies from Matthiessen et al. [19] and Ellebæk et al. [25] mainly focused on a demonstrated defect of the intestinal wall.

### 3.3. ELISA (Enzyme-Linked Immunosorbent Assay)

Although most studies used ELISA to determine the cytokine levels, different methods of measuring, handling, and storing the samples were used in the included studies (Table 4).

### 3.4. Peritoneal Cytokines

**Figure 2:**
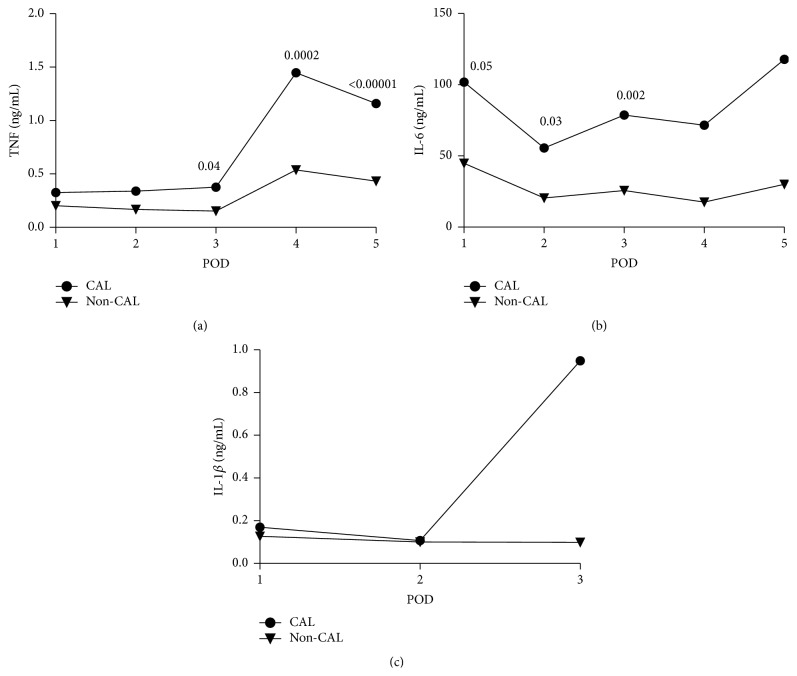
Weighted means of peritoneal levels of TNF (a, ng/mL), IL-6 (b, ng/mL), and IL-1*β* (c, ng/mL) on each postoperative day (POD) comparing colorectal anastomotic leakage (CAL) patients with non-CAL patients; TNF (a), IL-6 (b), and IL-1*β* (c). The *P* values of differences are illustrated when relevant.

In total we included 5 studies in the meta-analysis for peritoneal levels of TNF, Il-6, and IL-1*β*. The meta-analysis regarding peritoneal levels of cytokines included 228 patients who underwent colorectal surgery between 1996 and 2010. The mean level of cytokines on each postoperative day is reported in Figure 2 by calculating the weighted mean of each included study. As is shown in Figure 2 the peritoneal level of the cytokines varied after surgery. TNF and IL-6 levels substantially increased in patients with CAL while there was no or mild increase in patients without CAL.

**Figure 3:**
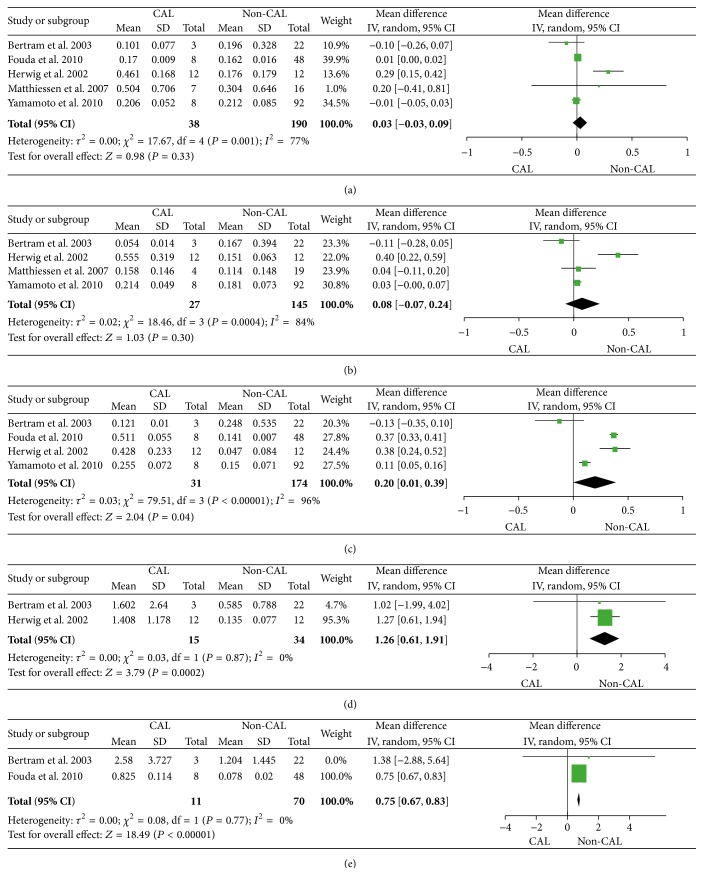
Forest plot with 95% confidence interval (CI) of the mean difference of peritoneal levels of TNF (ng/mL) between colorectal anastomotic leakage (CAL) patients and non-CAL patients per postoperative days (POD) 1 (a), 2 (b), 3 (c), 4 (d), and 5 (e).

**Figure 4:**
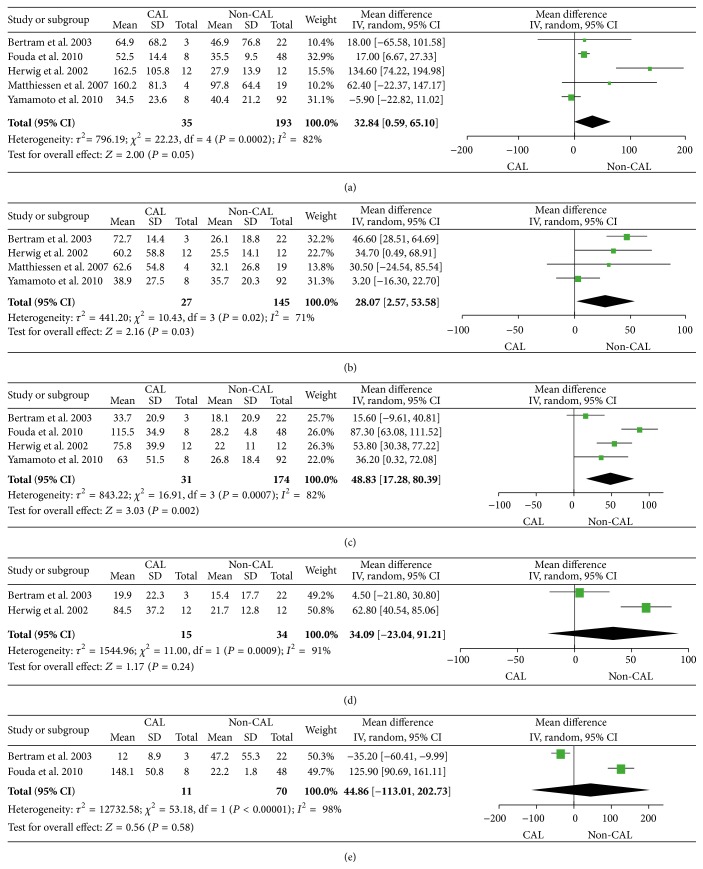
Forest plot with 95% confidence interval (CI) of the mean difference of peritoneal levels of IL-6 (ng/mL) between colorectal anastomotic leakage (CAL) patients and non-CAL patients per postoperative days (POD) 1 (a), 2 (b), 3 (c), 4 (d), and 5 (e).

Peritoneal levels of TNF showed significant differences between patients with and without CAL at POD3 (*P* = 0.04), POD4 (*P* = 0.0002), and POD5 (*P* < 0.00001) (Figure 3). The meta-analysis of POD3 included 4 studies while the meta-analyses of POD4 and POD5 only included 2 studies. Peritoneal levels of IL-6 were different between patients with and without the CAL on POD1 (*P* = 0.05), POD2 (*P* = 0.03), and POD3 (*P* = 0.002) (Figure 4). All analyses for IL-6 were based on 4 or 5 independent studies on the first three days. Nevertheless, peritoneal levels of IL-1*β* and IL-10 were only reported in one or two studies. Although we still performed the meta-analysis when possible, the results did not show significant differences between patients with and without CAL on each respective day (see Supplementary Material, Figure S1, available online at http://dx.doi.org/10.1155/2016/3786418).

### 3.5. Systemic Cytokines

Two studies were included for evaluation of systemic cytokine levels after colorectal surgery [25, 26]. Moreover, the primary data was not available in the articles. Therefore, we did not perform a meta-analysis for the systemic levels of cytokines. However, neither of the studies showed any significant difference in the systemic cytokines levels (TNF, IL-1*β*, IL-6, and IL-10) between patients with and without CAL.

## 4. Discussion

CAL remains a dangerous complication after colorectal surgery. This meta-analysis summarizes previous literature of early detection for CAL by measuring peritoneal and systemic cytokine levels. Our data show that peritoneal levels of proinflammatory cytokines (i.e., IL-6 and TNF) were higher in patients with CAL during early postoperative days, suggesting the diagnostic value of measuring the peritoneal cytokine level after surgery.

Among the candidate cytokines, TNF and IL-6 showed a statistically significant increase in CAL patients. These inflammatory cytokines are mainly secreted by macrophages and neutrophils, which infiltrate to the anastomotic area at the first days after construction of anastomosis. Our previous animal studies have demonstrated that a significantly larger amount of iNOS+ (Inducible Nitric Oxide Synthase) producing cells (mainly macrophages subtype 1) infiltrates into the anastomotic area in the CAL cases compared to those without CAL within the first postoperative days [29]. In accordance with previous evidence, current data confirm the localized mechanism during the early stage of CAL, suggesting the importance of these two cytokines, especially IL-6, in the early detection of CAL.

Nowadays the diagnosis of CAL still relies on clinical presentation and imaging studies. Early clinical presentation is often heterogeneous and nonspecific, resulting in delay of CAL diagnosis [9]. In many cases, CAL does not turn clinically apparent until approximately the eighth postoperative day [30]; in some cases, CAL may even become manifest until a median of the twelfth postoperative day when many patients have already been discharged [8]. As shown in our results, increased peritoneal levels of proinflammatory cytokines were observed on postoperative days 1–5, which is much earlier than the current median day of CAL diagnosis [8]. IL-6 was especially higher in CAL patients since the first postoperative day, indicating the possibility of early detection of CAL by monitoring of intraperitoneal cytokines. Such possibility has been reported by the previous literature. The studies from Salgado et al. [31, 32] reported that the increase of peritoneal cytokine levels is prior to the clinical manifestations of anastomotic leakage or increase of leukocytes in bariatric surgery. Similar investigations in the field of colorectal surgery are also warranted.

We also attempted to explore whether the systemic cytokine level could contribute to the early detection of CAL, since postoperative drainage is not often applied in colonic surgeries nowadays [33–35]. Measurement of systemic cytokines might be of great assistance to early detection of CAL in patients without drainage if their early changes can be determined as well. Unfortunately our data show that higher levels of cytokines were only observed in the peritoneal drainage but not in the blood sample. Despite the lack of high level-of-evidence studies, our findings are in line with the previous study from Wiik et al. who reported a more extensive release of pro- and anti-inflammatory cytokines into the peritoneal cavity after abdominal surgery compared to the systemic response [12]. This could be due to the fact that lymphocytes and monocytes at the site of CAL secrete these cytokines [36]. In addition, other studies have demonstrated a poor diagnostic value of serum CRP or white blood cell count in the early detection of CAL [37]. Accumulated evidence verified that the systematic changes of CAL are rather latent during the first postoperative days. Changes in systemic levels of cytokines seem to only occur when a critical condition emerges such as sepsis [38].

For the purpose of determining a reference level in colorectal surgery, we used exact values rather than the comparative risk ratios in statistical analysis, which was previously reported by Cini et al. [39]. According to our data, it seems that although many included studies reported a significant risk of CAL with high cytokine levels, the repeatability of the cytokine levels among different studies still seems unsatisfactory. The variations in cytokine levels may be caused by several reasons. As mentioned above, the definition of CAL varies substantially among the included studies (Table 3). The included articles used different definitions of CAL, which correspond to different grades of CAL according to the International Study Group on Rectal Cancer, varying between subclinical CAL to the ones requiring surgical intervention. This induced a mix of outcomes in this meta-analysis.

Due to the relatively low rate of infectious complications after colorectal surgery, studies with high level-of-evidence and a large amount of patients on such topic are difficult to implement. It is understandable that, in this early stage, most included studies yield very low level-of-evidence and high risks of bias. (Nested) case-control studies are highly sensitive to bias, especially to the selection bias, which may influence reliability of the study results. Moreover, the included studies have to deal with a limited sample size, which also decreases the reliability. On the basis of these limitations the studies are sensitive to the type II error (i.e., false negative). However this type II error has limited influence to the positive results of our analysis (for TNF and IL-6), supporting higher peritoneal cytokine levels in CAL patients compared to uncomplicated cases. Unfortunately, previous studies do not provide further data regarding the sensitivity and specificity of the peritoneal cytokine evaluation. Studies with high level-of-evidence and large amount of inclusions to determine the role of peritoneal cytokines in the early diagnosis of CAL are needed, which is also one main focus of our on-going study.

## 5. Conclusions

In this meta-analysis, we investigated both peritoneal and systemic cytokine levels after colorectal surgery. Our data demonstrate that levels of the peritoneal proinflammatory cytokines (i.e., TNF and IL-6) substantially increase in CAL patients during the first postoperative days, suggesting their potential diagnostic value, while the systemic cytokines have limited additional value in this regard. High level-of-evidence studies are warranted to determine the accuracy of peritoneal cytokines in the early diagnosis of CAL.

## Supplementary Material

Fig S1. Forest plot with 95% confidence interval (CI) of the mean difference of peritoneal levels of IL-1β (ng/mL) between anastomotic leakage (AL) patients and non-anastomotic (non-AL) leakage patients per postoperative day (POD) 1 (=a), 2 (=b) and 3 (=c). The results did not show significant differences between patients with and without CAL on each respective day.

## Figures and Tables

**Table 1:** Study characteristics of included studies, including Levels of Evidence 2011 according to the Centre for Evidence Based Medicine.

Author	Year	Patients	Study design	Location	Surgery type	Follow-up	Index test	Complication	Level-of-evidence
Bertram et al. [23]	2003	25	Case-control	Peritoneal	Colorectal surgery	7 days	TNF, IL6	CAL (*n* = 3)	4
Fouda et al. [21]	2011	56	Case-control	Peritoneal	Elective low anterior resection for rectal cancer	5 days	TNF, Il-6, IL-10	CAL (*n* = 8)	4
Herwig et al. [24]	2002	24	Case-control	Peritoneal	Colorectal surgery	4 days	TNF, IL-1B, IL6	CAL (*n* = 12)	4
Matthiessen et al. [4, 19]	2007	23	Case-control	Peritoneal	Anterior resection of the rectum for cancer	2 days	TNF, Il-6, IL-10	CAL (*n* = 7)	4
Yamamoto et al. [20]	2011	100	Case-control	Peritoneal	Elective resection for carcinoma of the sigmoid/rectum	3 days	TNF, IL-1B, IL6	Peritonitis (*n* = 8)	4
Ellebæk et al. [25]	2014	50	Case-control	Systemic	Low anterior resection for rectosigmoid cancer	5 days	TNF, IL-1B, IL6, IL10	CAL (*n* = 4)	4
Reisinger et al. [26]	2014	84	Case-control	Systemic	Colorectal surgery	7 days	IL6	CAL (*n* = 8)	4

**Table 2:** Quality assessment of the included studies by judging risk of bias and applicability using Quality Assessment of Diagnostic Accuracy Studies-2 (QUADAS-2). +: low risk of bias; −: high risk of bias; ?: not specified.

Author	Year	Risk of bias				Applicability		
		Patient Selection	Index test	Reference standard	Flow and timing	Patient selection	Index test	Reference standard
Bertram et al. [23]	2003	−	+	−	−	+	+	−
Fouda et al. [21]	2011	−	+	+	−	+	+	+
Herwig et al. [24]	2002	−	+	+	−	+	+	+
Matthiessen et al. [4, 19]	2007	−	+	+	−	+	+	+
Yamamoto et al. [20]	2011	−	+	+	−	+	+	+
Ellebæk et al. [25]	2014	−	+	?	−	+	+	?
Reisinger et al. [26]	2014	−	+	+	−	+	+	+

**Table 3:** Definition of anastomotic leakage of included studies; CAL: colorectal anastomotic leakage.

Author	Year	Complication	Definition
Bertram et al. [23]	2003	CAL	Patients were considered uneventful if recovery occurred without signs of anastomotic leakage within 14 days after operation
Fouda et al. [21]	2011	CAL	AL was defined clinically as gas, pus, or fecal discharge from the drain, fecal discharge from the operative wound, pelvic abscess, peritonitis, and rectovaginal fistula
Herwig et al. [24]	2002	CAL	Diagnosis of AL was confirmed by endoscopy, contrast enema, abdominal CT scan, microbiologic examination, and finally intraoperative findings during relaparotomy
Matthiessen et al. [4, 19]	2007	CAL	Peritonitis caused by leakage, pelvic abscess, discharge of feces from the abdominal drain, or rectovaginal fistula, and leakage from all staple lines
Yamamoto et al. [20]	2011	Peritonitis	The diagnosis of postoperative peritonitis was based on clinical findings along with imaginary data and the colour of abdominal exudates
Ellebæk et al. [25]	2014	CAL	Anastomotic leakage was defined as a demonstrated defect of the intestinal wall at the anastomotic site leading to a communication between the intra- and extraluminal compartment's
Reisinger et al. [26]	2014	CAL	Clinically relevant AL was defined as extra luminal presence of contrast fluid on contrast CT scans and/or leakage when relaparotomy was performed, requiring reintervention

**Table 4:** Specifying the methodology of cytokine level measurement of included studies. NS: not specified; ELISA: enzyme-linked immunosorbent assay.

Author	Year	Cytokines	Location	Centrifugation	Storage	Cytokine measuring	Producer
Bertram et al. [23]	2003	TNF, IL-6	Peritoneal	3000 rpm for 10 min at 4°C	−80°C	ELISA	Immulite, DPC Biermann GmbH, Bad Nauheim, Germany
Fouda et al. [21]	2011	TNF, IL-6, IL-10	Peritoneal	3000 rpm for 10 min at 4°C	20°C	ELISA	NS
Herwig et al. [24]	2002	TNF, IL-1*β*, IL-6	Peritoneal	2000 rpm for 10 min	−70°C	ELISA	Coulter-Immunotech Diagnostics, Hamburg, Germany
Matthiessen et al. [4, 19]	2007	TNF, IL-6, IL-10	Peritoneal	NS	NS	ELISA	DPC, Los Angeles, CA, USA
Yamamoto et al. [20]	2011	TNF, IL-1*β*, IL-6	Peritoneal	3000 rpm for 10 min	−80°C	ELISA	R&D system, Minneapolis, MN, USA
Ellebæk et al. [25]	2014	TNF, IL-1*β*, IL-6, IL-10	Systemic	3600 rpm for 10 min at 4°C	−80°C	ELISA	Bio-Rad Laboratories, Hercules, CA, USA
Reisinger et al. [26]	2014	IL-6	Systemic	3500 rpm for 15 min	−80°C	ELISA	NS
