# Supplementary material for: Cytokines as Early Markers of Colorectal Anastomotic Leakage: A Systematic Review and Meta-Analysis
Source: Gastroenterol Res Pract. 2016 Mar 9;2016:3786418. doi: 10.1155/2016/3786418 (PMC4804081; doi:10.1155/2016/3786418)
Supplement: Supplementary file 1 — Fig S1. Forest plot with 95% confidence interval (CI) of the mean difference of peritoneal levels of IL-1β (ng/mL) between anastomotic leakage (AL) patients and non-anastomotic (non-AL) leakage patients per postoperative day (POD) 1 (=a), 2 (=b) and 3 (=c). The results did not show significant differences between patients with and without CAL on each respective day. [file 3786418.f1.docx]

Supplementary data:

**
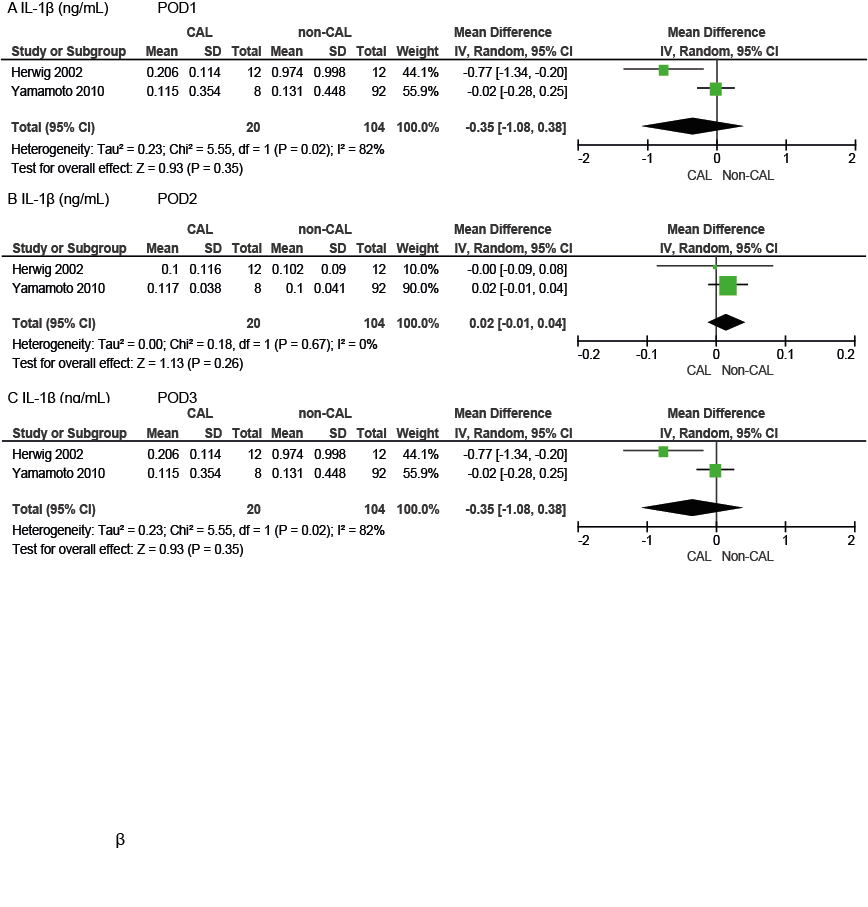
**

**Fig S1** Forest plot with 95% confidence interval (CI) of the mean difference of peritoneal levels of IL-1β (ng/mL) between colorectal anastomotic leakage (CAL) patients and non-CAL patients per postoperative day (POD) 1 (=A), 2 (=B) and 3 (=C).
